# Proportion of Fentanyl Reports in Illicit Drug Seizures and Opioid Mortality

**DOI:** 10.1001/jamahealthforum.2025.6286

**Published:** 2026-01-16

**Authors:** Alex Dahlen, Frederick Lei, Kofi Agyabeng, Runhan Chen, Christian E. Johnson, Gabriel Amaro, Jake Spinnler, Mehrdad Khezri, José A. Pagán, Cheryl Healton, Tilda M. Farhat

**Affiliations:** 1 Biostatistical Collaboration and Consultation Core, Department of Biostatistics, School of Global Public Health, New York University, New York; 2 Office of the National Drug Control Policy, Executive Office of the President, Washington, DC; 3 Department of Epidemiology, School of Global Public Health, New York University, New York; 4 Department of Public Health Policy and Management, School of Global Public Health, New York University, New York

## Abstract

**Importance:**

The monthly opioid overdose death rate in the US has declined by 50% from its peak in the summer of 2023 through fall of 2024, and the factors associated with this decline are not fully understood.

**Objective:**

To examine the association between the proportion of fentanyl reports in illicit drug seizures and opioid overdose deaths during periods of rising and falling mortality.

**Design and Setting:**

This secondary analysis of state-month level panel data from the National Forensic Laboratory Information System and US Centers for Disease Control and Prevention Wide-Ranging Online Data for Epidemiologic Research (CDC WONDER) was conducted from January 2018 to September 2024 and included all 50 US states and Washington, DC. CDC WONDER data were collected from recorded death certificates; National Forensic Laboratory Information System data were obtained from drug reports submitted by forensic laboratories. The data were analyzed from July to August 2025.

**Exposure:**

Percentage of illicit drug seizures that contained fentanyl or fentanyl-related compounds. Illicit drug seizures were defined as seizures that contained any of the following: fentanyl or fentanyl-related compounds, heroin, methamphetamine, cocaine, and xylazine.

**Main Outcomes and Measures:**

The monthly count of opioid overdose deaths, given by uniform claim descriptor codes X40 to 44, X60 to 64, X85, and Y10 to Y14, with additional multiple cause of death codes of T40.0 to 4 and T40.6. Death rates were calculated using yearly population estimates from the American Community Survey.

**Results:**

From a peak in the summer of 2023 through the fall of 2024, the monthly opioid overdose death rate declined by 50%, from 2.2 to 1.1 per 100 000. This decline was was accompanied by a decline in the fentanyl reports as a proportion of total illicit drug seizures from 28.8% to 23.2%. In a 2-way, fixed-effects model, a 1–percentage point reduction in fentanyl prevalence was associated with 0.018 fewer overdose deaths per 100 000 population per month (95% CI, 0.016-0.019; *P* < .001). There was evidence that the strength of this association has decreased over time.

**Conclusions and Relevance:**

The study results suggest that current decline in the proportion of fentanyl reports in illicit drug seizures is associated with 9.2% of the total observed decline in mortality. Additional contributing factors may include other shifts in the drug supply not captured by fentanyl prevalence in illicit drug seizures, shifts in drug use behavior, and the effect of public health programs, interventions, and policies.

## Key Points

**Question:**

Was the recent decline in opioid overdose deaths accompanied by a corresponding decline in the share of fentanyl reports in illicit drug seizures?

**Findings:**

In this time series analysis of national data from all 50 US states and Washington, DC, from the peaks in summer 2023 through fall 2024, the monthly opioid overdose death rate declined by 50% and fentanyl reports declined from 28.8% of illicit drug seizures to 23.2%. The association between the 2 trends was significant but modest compared with the overall decline in deaths and appeared to decline in magnitude with time.

**Meaning:**

These findings suggest that other factors are likely contributing to the decline in mortality.

## Teaser

This cohort study examines the association between the proportion of fentanyl reports in illicit drug seizures and opioid overdose deaths during periods of rising and falling mortality.

## Introduction

The monthly opioid overdose death rate in the US has decreased by nearly 50% from its peak of 2.2 deaths per 100 000 in summer 2023 through fall 2024. The timing and magnitude of the decline has varied across regions and demographic groups,^1^ and the underlying drivers remain unclear.

Prior research found an association between the proportion of fentanyl reports among total illicit drug seizures and overdose deaths.^2,3,4,5,6,7^ From 2018 to 2022, both indicators rose in parallel, following a well-documented geographic pattern, with fentanyl appearing on the East Coast around 2017 and spreading westward.^8,9^

In this time series analysis, we ask whether this association now holds in reverse: is declining mortality accompanied by a corresponding decline in fentanyl reports? We used the proportion of fentanyl reports in illicit drug seizures as a proxy for fentanyl in the drug supply and examined the association between fentanyl reports and opioid overdose deaths during periods of rising and falling mortality.

## Methods

Monthly opioid overdose mortality rates were obtained for all 50 US states and Washington, DC, from January 2018 to September 2024 using the US Centers for Disease Control and Prevention Wide-Ranging Online Data for Epidemiologic Research (CDC WONDER) database.^10^ This analysis of deidentified, publicly available data was determined exempt from institutional review board approval by the NYU Washington Square institutional review board under 45 CFR §46. This study followed the Enhancing the Quality and Transparency Of Health Research (EQUATOR) reporting guidelines. Drug overdose deaths were identified using *International Classification of Diseases, 10th Revision* (*ICD-10*) underlying cause of death codes X40 to X44, X60 to X64, X85, and Y10 to Y14. The primary outcome, opioid overdose deaths, was defined using multiple cause of death codes (T40.0-T40.4 and T40.6; deaths involving opium, heroin, other opioids, methadone, other synthetic narcotics, or unspecified narcotics). Secondary outcomes included (1) any/all drug overdose deaths and (2) synthetic opioid overdose deaths (T40.4 only), which are broader and narrower than the primary outcome, respectively. Death counts were transformed into rates per 100 000 using American Community Survey yearly population estimates. To address the suppression of small cells (1-9) in the CDC WONDER database, we evenly allocated the difference between the yearly deaths for a state and the unsuppressed monthly total across suppressed months; for sensitivity analyses, we imputed suppressed cells as 1 and 9.

Monthly law enforcement drug seizures data for the same period (January 2018 to September 2024) were extracted from the National Forensic Laboratory Information System, which captures approximately 98% of analyzed seizures nationwide.^11^ Entries correspond to documented seizures, regardless of quantity or potency, and are indexed to the date the seizure occurred. Following Cano et al,^2^ we used the fraction of seizures that involved fentanyl or fentanyl analogs among total seizures of illicit drugs (methamphetamine, cocaine, xylazine, heroin, carfentanil, or fentanyl) as a proxy for the proportion of fentanyl in the illicit drug supply. In effect, this treats drug seizures as a random sample of the drug supply within each state.

To estimate the association, we used a population-weighted, 2-way fixed-effects model of the form of *mortality_it_* = Σ_i_α_i_ + Σ_t_γ_t_ + β_1_*fentanyl_it_* + u_it_, in which *i* indexes states (and Washington DC), *t* indexes months, and *α_i_* and *γ_t_* are the state-level and month-level fixed-effects, respectively. We used 2-way clustered confidence intervals. We also fit versions with census division–fentanyl interactions and with period–fentanyl interactions. Three periods were considered: January 2018 to February 2020 (pre–COVID-19 pandemic, increasing mortality), March 2020 to December 2022 (during COVID-19 pandemic, increasing mortality), and January 2023 to September 2024 (post–COVID-19 pandemic, decreasing mortality); a sensitivity analysis considered yearly periods. We conducted several modeling sensitivity analyses: an unweighted version of the primary model, a Poisson regression model with population offsets, and versions of the model for the secondary outcomes (any/all drug overdose deaths and synthetic opioid overdose deaths). Analyses were conducted in R, version 4.5.1 (R Foundation), and statistical significance was set at 5%. The code is available,^12^ and; full statistical methods are described in the eMethods in Supplement 1.

## Results

**Figure 1.**
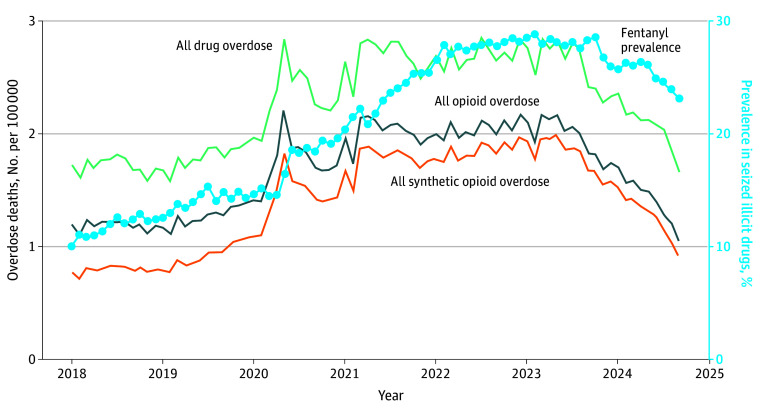
National Trend in Overdose Deaths per 100 000 per Month and the Proportion of Fentanyl in the Illicit Drug Supply From January 2018 to September 2024. Figure 2 shows the same trends by US Census division.

**Figure 2.**
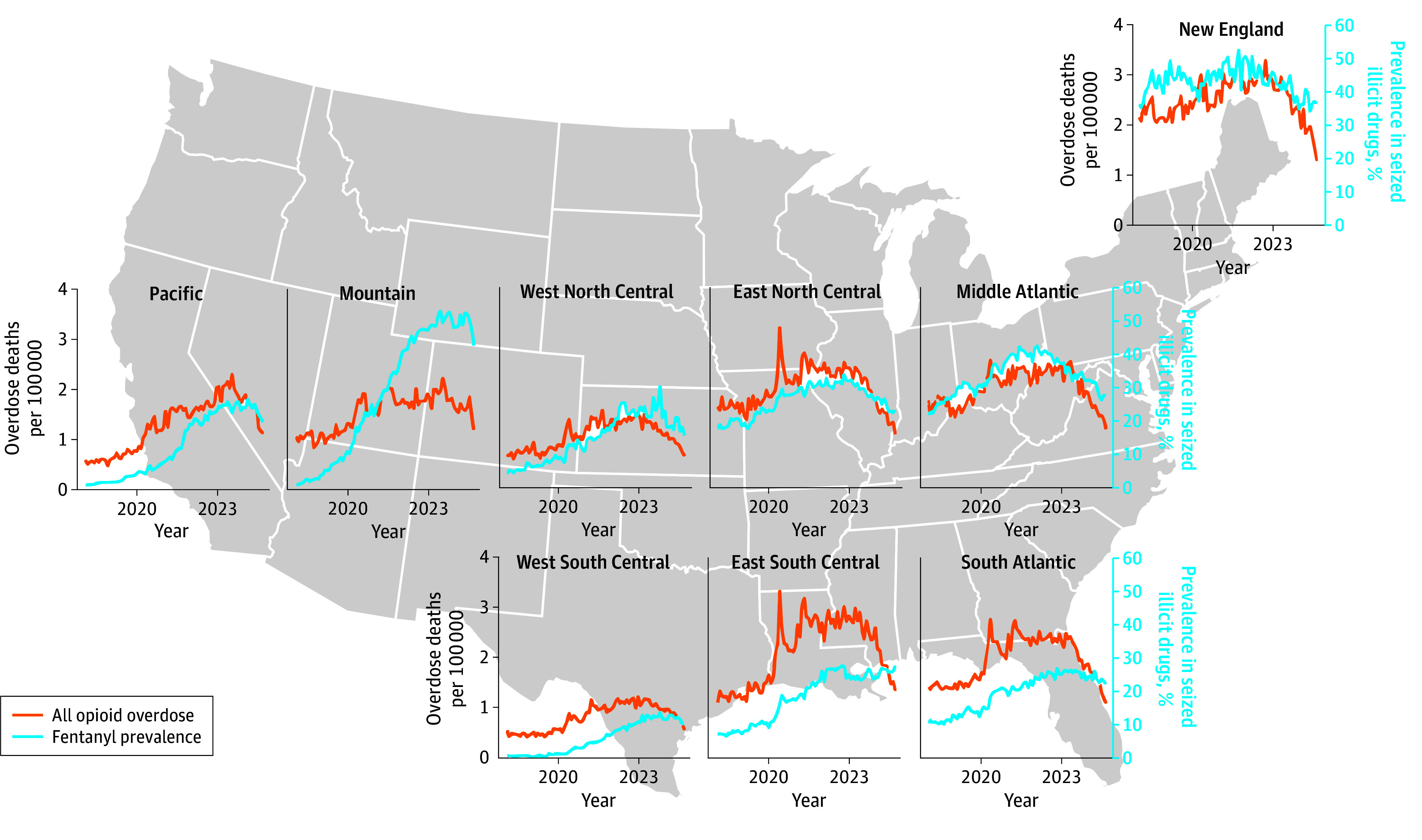
Trends in Opioid Overdose Deaths per 100 000 per Month and the Proportion of Fentanyl Reports Among Total Illicit Drug Seizures From January 2018 to September 2024; decomposed by US Census division.

Figure 1 and Figure 2 show the trends in overdose deaths and the proportion of fentanyl reports in illicit drug seizures nationally and by census division. Both trends grew steadily through the winter of 2022, when they peaked and begin declining. From winter 2022 to fall 2024, the monthly national opioid overdose death rate decreased by 50% (2.2 to 1.1 per 100 000), and the proportion of fentanyl decreased from 28.8% to 23.2%. The decline appeared to begin on the East Coast and travel westward.

**Figure 3.**
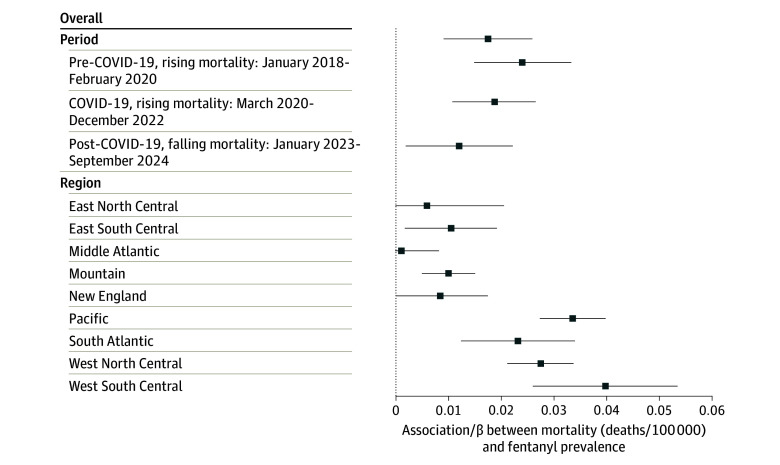
Two-Way Fixed-Effects Model Results for the Primary Outcome of Opioid Overdose Deaths Stratified by period and US Census division. The whiskers indicate 95% CIs.

We found an association between the monthly proportion of fentanyl reports in the illicit drug supply and overdose deaths (Figure 3). On average, a state with a 1–percentage point decrease in the proportion of fentanyl reports had 0.018 fewer deaths per 100 000 population per month (95% CI, 0.016-0.019). Therefore, the observed 5.6–percentage point decline in the share of fentanyl reports was associated with around 340 fewer deaths per month, 9.2% of the total observed decline. There was evidence of a decreasing magnitude of the association across the 3 periods and of larger associations in the western US. Results were similar for the secondary outcomes (eTable 1 in Supplement 1) and were consistent across modeling strategies (eTable 2 in Supplement 1), methods for addressing suppressed cells (eTable 3 in Supplement 1), and period specifications (eFigure 2 in Supplement 1).

## Discussion

In this time series analysis, we found an association between opioid overdose mortality and the proportion of fentanyl reports in law enforcement drug seizures. Between 2018 and 2024, both indicators rose, peaked, and started to decline, although the timing varied by region. The magnitude of the association was relatively small compared with the overall decline in deaths and appears to be decreasing over time. This was also evident when comparing the periods of rising and falling mortality: from 2018 to 2022, mortality doubled while the fentanyl reports in illicit drug seizures rose by 17 percentage points (from 12% to 29%). When mortality decreased by half from 2023 to 2024, fentanyl prevalence only fell by 5.6 percentage points.

The factors associated with the decline in the fentanyl reports as a proportion of illicit drug seizures are unclear. The 5.6–percentage point decrease in the fentanyl reports in 2024 coincided with a commensurate increase in the proportion of cocaine reports from 20.9% to 26.3% (eFigure 1 in Supplement 1). These trends could reflect shifts in preferences, increased awareness of risks, or education and prevention efforts; it could also reflect shifting enforcement priorities over time.

### Limitations

One reason for the modest association may be that our proxy indicator, which is based on law enforcement seizures, may not have fully captured key aspects of the drug supply. While widely used, this measure assumes that seizures represented a sample of the illicit drug market; in reality, enforcement patterns and seizure targeting vary across jurisdictions and over time. The proxy indicator also does not capture weight, purity, potency, form, or composition (carfentanil vs fentanyl with xylazine), and it excludes prescribed opioids. If any of these factors change over time within states, it could have introduced bias in our results. Therefore, these estimates should be interpreted with caution and viewed as approximations of broader market trends.

Beyond supply-related factors, the recent decline in mortality may also reflect changing user preferences (eg, smoking vs injecting) or the effect of public health, health care, and community-based interventions. Programs such as naloxone distribution, Good Samaritan laws, prescription drug monitoring programs, overdose prevention efforts, and increased access to medications for opioid use disorder expanded during the study period.^13,14,15,16^ Wider adoption of these initiatives may explain the apparent decrease in the association during the 3 periods.

## Conclusions

This time series analysis found an association between opioid overdose mortality and the proportion of fentanyl reports in law enforcement drug seizures. The findings suggest that the decline in overdose mortality reflects a combination of changing drug supply dynamics and evolving public health and policy responses.

## Notes

Supplement 1. eMethods. eFigure 1. The trend in total illicit drug seizures, and the makeup of different illicit drugs eTable 1. The association between fentanyl prevalence in illicit drug seizures and three measures of mortality eTable 2. Results of our two modeling sensitivity analyses for the primary outcome of opioid overdose deaths eTable 3. Results of two sensitivity analyses that impute suppressed death count cell sizes by either all 1’s or all 9’s eFigure 2. Results of a sensitivity analysis that consider yearly time periods instead of the 3 time periods in our primary model eTable 4. Number of months with suppressed data for each year, by state and mortality outcome

Supplement 2. Data sharing statement
