# Supplementary material for: Proportion of Fentanyl Reports in Illicit Drug Seizures and Opioid Mortality
Source: JAMA Health Forum. 2026 Jan 16;7(1):e256286. doi: 10.1001/jamahealthforum.2025.6286 (PMC12811803; doi:10.1001/jamahealthforum.2025.6286)
Supplement: Supplement 1. — eMethods. eFigure 1. The trend in total illicit drug seizures, and the makeup of different illicit drugs eTable 1. The association between fentanyl prevalence in illicit drug seizures and three measures of mortality eTable 2. Results of our two modeling sensitivity analyses for the primary outcome of opioid overdose deaths eTable 3. Results of two sensitivity analyses that impute suppressed death count cell sizes by either all 1’s or all 9’s eFigure 2. Results of a sensitivity analysis that consider yearly time periods instead of the 3 time periods in our primary model eTable 4. Number of months with suppressed data for each year, by state and mortality outcome [file jamahealthforum-e256286-s001.pdf]

Supplemental Online Content

Dahlen A, Lei F, Agyabeng K. Proportion of fentanyl reports in illicit drug seizures and opioid mortality. *JAMA Health Forum*. 2026;7(1):e256286.
doi:10.1001/jamahealthforum.2025.6286

eMethods.
eFigure 1. The trend in total illicit drug seizures, and the makeup of different illicit drugs
eTable 1. The association between fentanyl prevalence in illicit drug seizures and three measures of mortality
eTable 2. Results of our two modeling sensitivity analyses for the primary outcome of opioid overdose deaths
eTable 3. Results of two sensitivity analyses that impute suppressed death count cell sizes by either all 1's or all 9's
eFigure 2. Results of a sensitivity analysis that consider yearly time periods instead of the 3 time periods in our primary model
eTable 4. Number of months with suppressed data for each year, by state and mortality outcome

This supplemental material has been provided by the authors to give readers additional information about their work.

© 2026 Dahlen A et al.*JAMA Health Forum*.

eMethods. CDC Wonder data suppresses death counts between 1 and 9. Our primary method of imputing values for the suppressed cells was to compute the (unsuppressed) yearly death count in that state, and to distribute the unattributed deaths evenly across months with suppressed cells. In particular, if for a given state and year, if there was only one month with a suppressed cell, this process produces an unambiguous result: the unattributed deaths (the yearly death count minus the sum of the 11 months with unsuppressed counts) necessarily equals the value in the suppressed cells. If two or more months have suppressed cells, we distribute the unattributed deaths evenly between the months with suppressed counts. This method keeps the total death count by state and year correct. As sensitivity analyses, we also consider imputing all 1's for the suppressed cells and all 9's for the suppressed cells, which are under- and over-estimates of the total. The count of the number of suppressed cells by state and year for all 3 metrics is shown in **eTable 6.**

Our primary statistical model is a weighted TWFE regression of the form:

$$\text{mortality}_{it} = \sum_{i=1}^{51} \alpha_i + \sum_{t=1}^{81} \gamma_t + \beta_1 \text{fentanyl}_{it} + u_{it},$$

where i indexes states (and DC), and t indexes months. There are 51 state-level fixed effects α_i and 81 month-level fixed effects γ_t. Data are weighted by the state population, and we use two-way clustered confidence intervals. Results for the primary outcome of opioid overdose deaths, as well as for the secondary outcomes of any/all drug overdose deaths and synthetic opioid overdose deaths are shown in **eTable 1**.

We interrogated the robustness of these results with several sensitivity analyses. To test for robustness to modeling decisions, we fit two additional versions of the model for the primary outcome. The first is an unweighted version, and the second is a weighted Poisson regression with deaths treated as count data and population as an offset. Results of these sensitivity analyses are shown in **eTable 2.** To test for robustness to our strategy for imputing suppressed cells, we report versions of the primary result with the all-1's and all-9's imputation strategies in **eTable 3.**

To explore potential heterogeneity in the size of the association (β_1), we also fit a model with census division-fentanyl interactions, and one with time period-fentanyl interactions. Three time periods were considered: January 2018 to February 2020 (pre-COVID, rising mortality), March 2020 to December 2022 (COVID, rising mortality), and January 2023 to September 2024 (post-COVID, falling mortality), and as a sensitivity analysis, we also considered yearly time periods. The results of this model are shown in **eTable 4.**

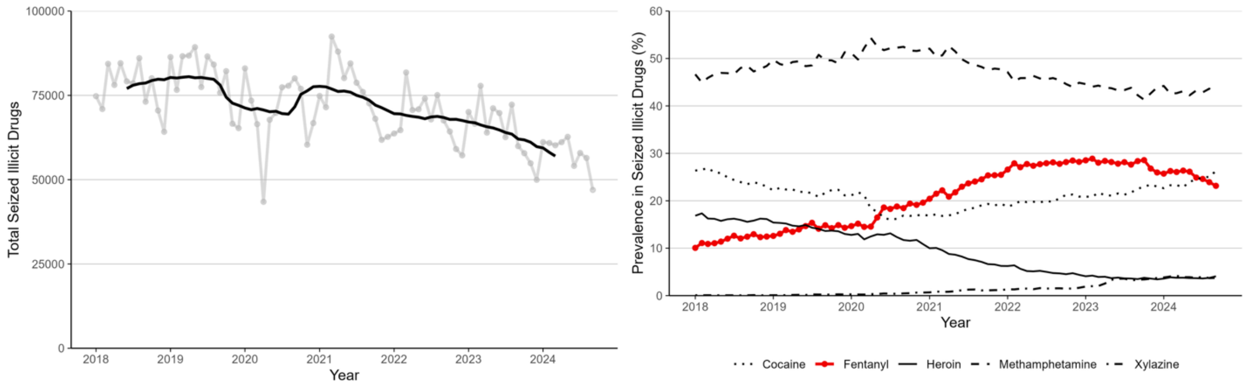

eFig 1. Left: The trend in total illicit drug seizures which has been mostly stable over time. There is a dip associated with the early months of the COVID-19 pandemic, and a slight decline over time. Right: the makeup of different illicit drugs. The lines sum to 100%. While fentanyl was increasing in the drug supply (2018-2022), it was mostly replacing heroin; the current decline in fentanyl is mostly from a rise in cocaine

© 2026 Dahlen A et al. *JAMA Health Forum*.

	All Opioid Overdose Deaths	Any/All Drug Overdose Deaths	All Synthetic Opioid Deaths
	β (std. err.)	β (std. err.)	β (std. err.)
Overall	0.0176*** (0.0042)	0.0190*** (0.0047)	0.0221*** (0.0043)
Time period			
Jan 2018 - Feb 2020	0.0241*** (0.0047)	0.0285*** (0.0050)	0.0286*** (0.0047)
Mar 2020 - Dec 2022	0.0188*** (0.0041)	0.0198*** (0.0045)	0.0235*** (0.0043)
Jan 2023 - Sep 2024	0.0121* (0.0052)	0.0125* (0.0055)	0.0164** (0.0054)
Census division			
East North Central	0.0060 (0.0075)	0.0037 (0.0088)	0.0097 (0.0087)
East South Central	0.0418*** (0.0052)	0.0448*** (0.0062)	0.0475*** (0.0061)
Middle Atlantic	0.0076 (0.0131)	0.0033 (0.0136)	0.0099 (0.0130)
Mountain	0.0102*** (0.0026)	0.0118*** (0.0033)	0.0159*** (0.0029)
New England	0.0086+ (0.0046)	0.0088+ (0.0052)	0.0106* (0.0041)
Pacific	0.0336*** (0.0032)	0.0366*** (0.0035)	0.0376*** (0.0032)
South Atlantic	0.0232*** (0.0055)	0.0254*** (0.0061)	0.0279*** (0.0061)
West North Central	0.0106* (0.0044)	0.0094* (0.0042)	0.0101* (0.0043)

***p<0.001, **p<0.01, *p<0.05, +p<0.1

eTable 1: The association between fentanyl prevalence in illicit drug seizures and three measures of mortality: opioid overdose deaths, any/all drug overdose deaths (strictly larger) and synthetic opioid overdose deaths (strictly smaller). We provide overall effect sizes, as well as estimates for time period- and region-specific effect sizes from versions of the model with interaction terms. The all opioid overdose deaths column corresponds to the model results **Fig. 3**.

	Unweighted TWFE OLS	TWFE Poisson	
	β (std. err.)	β (std. err.)	IRR
Overall	0.0133*** (0.0033)	0.0094*** (0.0025)	1.009
Time period	0.0144** (0.0053)	0.0153*** (0.0027)	1.015
Jan 2018 - Feb 2020	0.0160*** (0.0032)	0.0051** (0.0017)	1.005
Mar 2020 - Dec 2022	0.0097* (0.0038)	0.0041+ (0.0022)	1.004
Jan 2023 - Sep 2024	0.0144** (0.0053)	0.0153*** (0.0027)	1.015
Census division			
East North Central	0.0025 (0.0067)	-0.0024 (0.0029)	0.998
East South Central	0.0352*** (0.0064)	0.0136*** (0.0026)	1.014
Middle Atlantic	-0.0044 (0.0136)	-0.0051 (0.0076)	0.995
Mountain	0.0079** (0.0026)	0.0055** (0.0020)	1.006
New England	0.0084+ (0.0044)	-0.0009 (0.0021)	1.000
Pacific	0.0322*** (0.0060)	0.0229*** (0.0034)	1.023
South Atlantic	0.0170* (0.0081)	0.0058* (0.0026)	1.006
West North Central	0.0029 (0.0034)	0.0125* (0.0051)	1.013

***p<0.001, **p<0.01, *p<0.05, +p<0.1

eTable 2: Results of our two modeling sensitivity analyses for the primary outcome of opioid overdose deaths. The unweighted TWFE OLS model has the same structure as our primary model except that it is not population-weighted. The TWFE Poisson model uses mortality counts as the outcome and the log of the population estimate as an offset. Results are broadly consistent across modeling strategies.

© 2026 Dahlen A et al.*JAMA Health Forum*.

	Evenly distribute “unattributed deaths” (primary method)	All suppressed cells are 1	All suppressed cells are 9
	β (std. err.)	β (std. err.)	β (std. err.)
Overall	0.0176*** (0.0042)	0.0177*** (0.0042)	0.0174*** (0.0042)
Time period			
Jan 2018 - Feb 2020	0.0241*** (0.0047)	0.0241*** (0.0048)	0.0237*** (0.0048)
Mar 2020 - Dec 2022	0.0188*** (0.0041)	0.0189*** (0.0041)	0.0188*** (0.0041)
Jan 2023 - Sep 2024	0.0121* (0.0052)	0.0122* (0.0052)	0.0119* (0.0052)
Census division			
East North Central	0.0418*** (0.0052)	0.0417*** (0.0051)	0.0420*** (0.0052)
East South Central	0.0336*** (0.0032)	0.0338*** (0.0033)	0.0335*** (0.0032)
Middle Atlantic	0.0102*** (0.0026)	0.0102*** (0.0026)	0.0099*** (0.0026)
Mountain	0.0397*** (0.0070)	0.0396*** (0.0070)	0.0401*** (0.0069)
New England	0.0086+ (0.0046)	0.0091+ (0.0047)	0.0085+ (0.0046)
Pacific	0.0232*** (0.0055)	0.0231*** (0.0055)	0.0235*** (0.0055)
South Atlantic	0.0060 (0.0075)	0.0059 (0.0075)	0.0063 (0.0075)
West North Central	0.0106* (0.0044)	0.0101* (0.0047)	0.0097+ (0.0050)

***p<0.001, **p<0.01, *p<0.05, +p<0.1

eTable 3: Results of two sensitivity analyses that impute suppressed death count cell sizes by either all 1’s or all 9’s, for the primary outcome of opioid overdose deaths (2nd and 3rd columns), as well as for our primary method of evenly distributing “unattributed” deaths (1st column). Results for the magnitude of the associations are broadly consistent with our primary TWFE estimator.

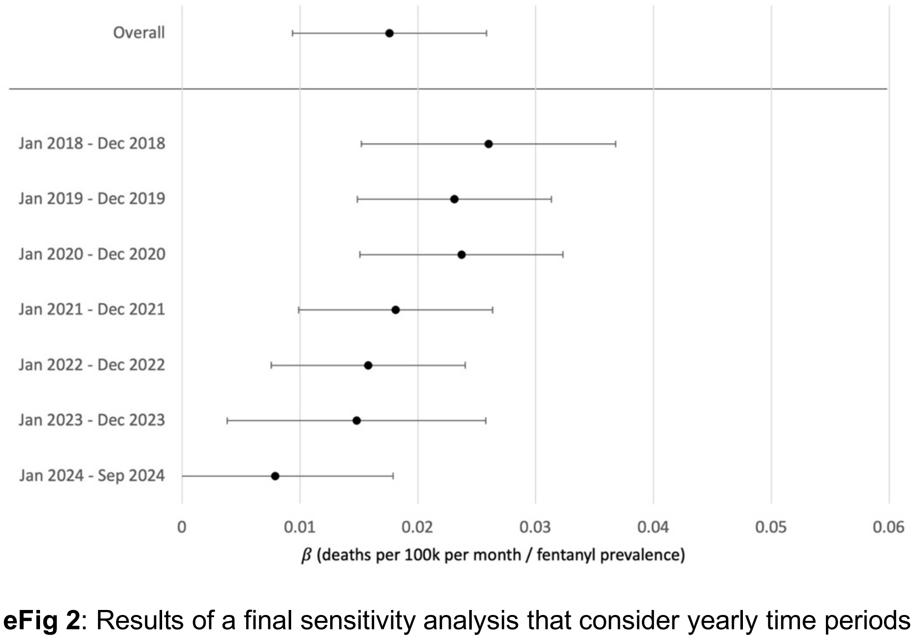

eFig 2: Results of a final sensitivity analysis that consider yearly time periods (Jan 2018-Dec 2018, Jan 2019-Dec 2018, etc.) instead of the 3 time periods in our primary model (**Fig. 3**). Note that because our data ends in Sep 2024, the data from 2024 includes only 9 months. The decrease in the magnitude of the association between the proportion of fentanyl reports and opioid overdose deaths over time is evident.

© 2026 Dahlen A et al.*JAMA Health Forum*.

State	Type	2018	2019	2020	2021	2022	2023	2024
Alabama	Synthetic Opioid overdose death	2(16.7%)	0(0.0%)	0(0.0%)	0(0.0%)	0(0.0%)	0(0.0%)	0(0.0%)
Alaska	Drug overdose death	7(58.3%)	5(41.7%)	2(16.7%)	0(0.0%)	0(0.0%)	0(0.0%)	0(0.0%)
Alaska	All Opioid overdose death	11(91.7%)	8(66.7%)	9(75.0%)	0(0.0%)	0(0.0%)	0(0.0%)	0(0.0%)
Alaska	Synthetic Opioid overdose death	9(75.0%)	10(83.3%)	11(91.7%)	3(25.0%)	2(16.7%)	0(0.0%)	0(0.0%)
Arkansas	All Opioid overdose death	0(0.0%)	0(0.0%)	0(0.0%)	0(0.0%)	0(0.0%)	0(0.0%)	0(0.0%)
Arkansas	Synthetic Opioid overdose death	8(66.7%)	6(50.0%)	0(0.0%)	0(0.0%)	0(0.0%)	0(0.0%)	0(0.0%)
Colorado	Synthetic Opioid overdose death	3(25.0%)	0(0.0%)	0(0.0%)	0(0.0%)	0(0.0%)	0(0.0%)	0(0.0%)
Hawaii	Drug overdose death	0(0.0%)	0(0.0%)	0(0.0%)	0(0.0%)	0(0.0%)	0(0.0%)	0(0.0%)
Hawaii	All Opioid overdose death	11(91.7%)	10(83.3%)	10(83.3%)	9(75.0%)	8(66.7%)	5(41.7%)	3(33.3%)
Hawaii	Synthetic Opioid overdose death	9(75.0%)	8(66.7%)	11(91.7%)	11(91.7%)	10(83.3%)	7(58.3%)	5(55.6%)
Idaho	All Opioid overdose death	5(41.7%)	3(25.0%)	0(0.0%)	0(0.0%)	0(0.0%)	0(0.0%)	0(0.0%)
Idaho	Synthetic Opioid overdose death	11(91.7%)	10(83.3%)	11(91.7%)	3(25.0%)	0(0.0%)	0(0.0%)	2(22.2%)
Iowa	All Opioid overdose death	2(16.7%)	0(0.0%)	0(0.0%)	0(0.0%)	0(0.0%)	0(0.0%)	0(0.0%)
Iowa	Synthetic Opioid overdose death	11(91.7%)	9(75.0%)	2(16.7%)	0(0.0%)	2(16.7%)	0(0.0%)	3(33.3%)
Kansas	Synthetic Opioid overdose death	11(91.7%)	10(83.3%)	3(25.0%)	0(0.0%)	0(0.0%)	0(0.0%)	0(0.0%)
Mississippi	All Opioid overdose death	2(16.7%)	0(0.0%)	0(0.0%)	0(0.0%)	0(0.0%)	0(0.0%)	0(0.0%)
Mississippi	Synthetic Opioid overdose death	10(83.3%)	4(33.3%)	0(0.0%)	0(0.0%)	0(0.0%)	0(0.0%)	0(0.0%)
Montana	Drug overdose death	4(33.3%)	3(25.0%)	0(0.0%)	0(0.0%)	0(0.0%)	0(0.0%)	0(0.0%)
Montana	All Opioid overdose death	12(100.0%)	10(83.3%)	8(66.7%)	6(50.0%)	4(33.3%)	4(33.3%)	6(66.7%)
Montana	Synthetic Opioid overdose death	6(50.0%)	8(66.7%)	11(91.7%)	9(75.0%)	6(50.0%)	9(75.0%)	8(88.9%)
Nebraska	Drug overdose death	4(33.3%)	0(0.0%)	0(0.0%)	0(0.0%)	0(0.0%)	0(0.0%)	0(0.0%)
Nebraska	All Opioid overdose death	12(100.0%)	12(100.0%)	8(66.7%)	7(58.3%)	4(33.3%)	12(100.0%)	9(100.0%)
Nebraska	Synthetic Opioid overdose death	11(91.7%)	12(100.0%)	10(83.3%)	10(83.3%)	8(66.7%)	12(100.0%)	9(100.0%)
Nevada	Synthetic Opioid overdose death	10(83.3%)	8(66.7%)	0(0.0%)	0(0.0%)	0(0.0%)	0(0.0%)	0(0.0%)
New Mexico	Synthetic Opioid overdose death	8(66.7%)	2(16.7%)	0(0.0%)	0(0.0%)	0(0.0%)	0(0.0%)	0(0.0%)
North Dakota	Drug overdose death	10(83.3%)	10(83.3%)	7(58.3%)	5(41.7%)	3(25.0%)	4(33.3%)	3(33.3%)
North Dakota	All Opioid overdose death	11(91.7%)	12(100.0%)	11(91.7%)	11(91.7%)	8(66.7%)	10(83.3%)	9(100.0%)
North Dakota	Synthetic Opioid overdose death	6(50.0%)	7(58.3%)	12(100.0%)	12(100.0%)	9(75.0%)	11(91.7%)	9(100.0%)
Oklahoma	Synthetic Opioid overdose death	11(91.7%)	10(83.3%)	2(16.7%)	0(0.0%)	0(0.0%)	0(0.0%)	0(0.0%)
Oregon	Synthetic Opioid overdose death	7(58.3%)	8(66.7%)	0(0.0%)	0(0.0%)	0(0.0%)	0(0.0%)	0(0.0%)
Rhode Island	Synthetic Opioid overdose death	0(0.0%)	0(0.0%)	0(0.0%)	0(0.0%)	0(0.0%)	0(0.0%)	0(0.0%)
South Dakota	Drug overdose death	11(91.7%)	11(91.7%)	11(91.7%)	7(58.3%)	10(83.3%)	8(66.7%)	6(66.7%)
South Dakota	All Opioid overdose death	11(91.7%)	12(100.0%)	11(91.7%)	11(91.7%)	11(91.7%)	12(100.0%)	9(100.0%)
South Dakota	Synthetic Opioid overdose death	7(58.3%)	10(83.3%)	11(91.7%)	10(83.3%)	12(100.0%)	12(100.0%)	8(88.9%)

© 2026 Dahlen A et al. *JAMA Health Forum*.

Utah	Synthetic Opioid overdose death	9(75.0%)	7(58.3%)	2(16.7%)	0(0.0%)	0(0.0%)	0(0.0%)	0(0.0%)
Vermont	Drug overdose death	3(25.0%)	5(41.7%)	0(0.0%)	0(0.0%)	0(0.0%)	0(0.0%)	0(0.0%)
Vermont	All Opioid overdose death	4(33.3%)	5(41.7%)	3(25.0%)	0(0.0%)	0(0.0%)	0(0.0%)	0(0.0%)
Vermont	Synthetic Opioid overdose death	7(58.3%)	7(58.3%)	4(33.3%)	0(0.0%)	0(0.0%)	0(0.0%)	0(0.0%)
Wyoming	Drug overdose death	12(100.0%)	10(83.3%)	7(58.3%)	8(66.7%)	4(33.3%)	4(33.3%)	3(33.3%)
Wyoming	All Opioid overdose death	12(100.0%)	12(100.0%)	12(100.0%)	12(100.0%)	11(91.7%)	10(83.3%)	8(88.9%)
Wyoming	Synthetic Opioid overdose death	8(66.7%)	9(75.0%)	12(100.0%)	12(100.0%)	11(91.7%)	12(100.0%)	7(77.8%)

eTable 4. Number of months with suppressed data for each year, by state and mortality outcome. States with no suppressed data are not represented; these are: Arizona, California, Connecticut, Delaware, District of Columbia, Florida, Georgia, Illinois, Indiana, Kentucky, Louisiana, Maine, Maryland, Massachusetts, Michigan, Minnesota, Missouri, New Hampshire, New Jersey, New York, North Carolina, Ohio, Pennsylvania, South Carolina, Tennessee, Texas, Virginia, Washington, West Virginia, and Wisconsin. The total study period was 81 months. CDC-Wonder suppresses cell counts of between 1 to 9 deaths.

© 2026 Dahlen A et al.*JAMA Health Forum*.
