# Supplementary material for: Proportion of Fentanyl Reports in Illicit Drug Seizures and Opioid Mortality
Source: JAMA Health Forum. 2026 Jan 16;7(1):e256286. doi: 10.1001/jamahealthforum.2025.6286 (PMC12811803; doi:10.1001/jamahealthforum.2025.6286)
Supplement: Supplement 2. — Data sharing statement [file jamahealthforum-e256286-s002.pdf]

Data Sharing Statement

Dahlen. Proportion of Fentanyl Reports in Illicit Drug Seizures and Opioid Mortality. *JAMA Health Forum*. Published January 16, 2026. doi:10.1001/jamahealthforum.2025.6286

Data
Data available: No

Additional Information
Explanation for why data not available: Monthly drug seizure data made available by the ONDCP for this project cannot be shared, but data with quarterly aggregation are publicly available. The mortality data we used are publicly available from CDC Wonder.
